# Editorial: Proceedings and predictions in cardiac amyloidosis: unsolved mysteries and challenges for the future

**DOI:** 10.3389/fmed.2023.1232212

**Published:** 2023-07-17

**Authors:** Aldostefano Porcari, Gianfranco Sinagra, Julian D. Gillmore

**Affiliations:** ^1^Division of Medicine, National Amyloidosis Centre, University College London, London, United Kingdom; ^2^Centre for Diagnosis and Treatment of Cardiomyopathies, Cardiovascular Department, Azienda Sanitaria Universitaria Giuliano-Isontina (ASUGI), University of Trieste, Trieste, Italy; ^3^ European Reference Network for RARE, Low Prevalence and Complex Diseases of the Heart (ERN GUARD-Heart)

**Keywords:** amyloidosis, transthyretin, light chain (AL) amyloidosis, prognosis, treatments

*“Sic parvis magna” – Sir Francis Drake*

Perceptions of amyloidosis have changed dramatically over the recent years following major advances in diagnosis and therapeutic strategies (1), especially in the field of cardiac amyloidosis (2). Recognition of disease and confirmation of final diagnosis, clinical management and treatment approaches represent ongoing challenges (3). The article Research Topic on “*Proceedings and predictions in cardiac amyloidosis: unsolved mysteries and challenges for the future*” has been conceived to discuss the latest advances and gray areas to address (4, 5) in amyloidosis from the perspective of international experts in the field.

Cardiac amyloidosis is commonly caused by systemic light chain amyloidosis or transthyretin amyloidosis (6). Systemic light chain amyloidosis is an acquired condition which is caused by a clonal plasma or B-cell expansion (monoclonal gammopathy) producing amyloidogenic immunoglobulin (Ig) light chains (7). Transthyretin amyloidosis is caused by deposition of misfolded or cleaved transthyretin protein in organs. This condition may be hereditary, in presence of destabilizing genetic mutations, or non-hereditary (wild type form) in the absence of genetic mutations (1, 8). Advances in cardiac magnetic resonance imaging and cardiac scintigraphy with bone tracers have revolutionized the diagnosis of cardiac amyloidosis (9, 10). Porcari et al. address the change in epidemiology of light chain and transthyretin cardiac amyloidosis following the development of non-invasive diagnostic criteria, highlighting the difference in patient characteristics at presentation and outcome compared to the past. Receiving a diagnosis of cardiac amyloidosis or carrying a gene mutation with the associated risk of developing disease in the future has a psychological impact on patients and their families, which is investigated (Smorti et al.).

With the exception of transthyretin cardiac amyloidosis which can now be diagnosed non-invasively in ~70% of cases, histological analysis remains the definitive method for diagnosis and typing of amyloid (11). Clinical decision-making relies on histological information to provide patient-tailored clinical management, which includes Congo red staining used in combination with polarized light microscopy and immunohistochemistry (6), along with a mass spectrometry-based proteomic approach for amyloid typing (12). Riefolo et al. provide a thoughtful review on the contemporary histological approach to amyloidosis, with a focus on endomyocardial biopsy. The essential contribution of histology for typing amyloid fibril proteins is discussed in depth, especially in rare forms and challenging scenarios to orient treatment strategies.

Although histological studies reports a remarkable prevalence of disease (6), the epidemiology of amyloidosis, especially cardiac amyloidosis, is unknown worldwide (13). Caponetti et al. provide a comprehensive perspective on the association between underlying cardiac amyloidosis and specific clinical setting, potentially serving as tool to redefine screening approaches to amyloidosis in the real world (13). The authors underline the unique contribution of carpal tunnel syndrome that can precede the diagnosis of cardiac amyloidosis by 5 to 9 years (14). Among patients with bilateral surgery and ventricular thickening, the prevalence of transthyretin cardiac amyloidosis ranges from 14% in patients ≥60 years without occupational risk factors, to 33% considering only men (13, 15, 16). The authors emphasize how the adoption of sex-specific criteria to define cardiac amyloidosis might potentially result in earlier diagnoses (17, 18), and they discuss the results of a national survey of prevalence and accuracy of echocardiographic red flags of cardiac amyloidosis in consecutive patients undergoing routine echocardiography (AC-TIVE study) (19, 20), demonstrating a prevalence of transthyretin cardiac amyloidosis of 1% in the Italian population. Echocardiography is essential to raise suspicion of cardiac amyloidosis and provides multi-parametric assessment of systolic and diastolic cardiac. However, The value of left ventricle ejection fraction is limited in the setting of cardiac amyloidosis as discussed by Matteo et al.. The authors underline the potential advantages of other parameters for assessing cardiac contraction such as stroke volume (21), which might be an earlier marker of systolic dysfunction.

Cardiac amyloidosis has been traditionally considered an invariably fatal disease, but the scenario has changed following the development of disease-modifying treatments. The contemporary clinical course of transthyretin cardiac amyloidosis is characterized by progressive heart failure and arrhythmias (22). Scirpa et al. and Razvi et al. summarize the strengths and limitations of the main disease-specific staging systems developed to estimate survival and address the gray area of pre-symptomatic stage (23, 24) and foresee that a multi-parametric approach will result in a more accurate risk prediction (25). Tomasoni et al. address the advances in therapeutic strategies aimed at reducing the deposition of transthyretin amyloid in organs or hepatic synthesis of transthyretin through (26):

- Stabilization of the transthyretin tetramer to prevent dissociation (27, 28);
- Disruption of the messenger RNA (mRNA) in the hepatocyte with either small interfering RNA (29) or antisense oligonucleotides (30);
- Transthyretin gene editing to prevent the production of the relevant mRNA (31);
- Recognition of the transthyretin fibrils by the immune system and acceleration of their removal from vital organs with monoclonal antibodies (1);

Quarta et al. provides an expert opinion on the latest anti-fibril therapy in both light chain and transthyretin amyloidosis, which holds great potential for the application of depletion therapy in the foreseeable future.

Although amyloidosis is considered a contraindication to cardiac transplant, partly due to a perceived risk of amyloid recurrence in the allograft, cardiac transplant is a concrete therapeutic option for patients with light chain and transthyretin amyloidosis. Razvi et al. provide novel finding that “*Cardiac transplant is well-tolerated, restores functional capacity and improves prognosis in transthyretin cardiac amyloidosis. Post-cardiac transplant 1-year survival was 100%, 3-year survival was 92%, and 5-year survival was 90%. All but one surviving patient were New York Heart Association functional class I. Bone scintigraphy did not show evidence of cardiac allograft amyloid infiltration at 12 years post cardiac transplant*.” Notably, the authors provide the first evidence that the risk of amyloid recurrence in the cardiac allograft is low, thus suggesting that transthyretin amyloidosis does not represent a contraindication to cardiac transplant and that this treatment option should not be denied in patients with feasible characteristics. Future dedicated research is required to select the best candidates to cardiac transplant.

The epidemiology of cardiac amyloidosis is rapidly changing due to enhanced recognition of disease and the possibility of non-invasive diagnosis for transthyretin amyloidosis. Many gray areas have to be addressed (32). An exciting horizon of possibilities awaits to be explored in amyloidosis.

## Author contributions

AP, GS, and JG contributed to the conception, design, manuscript preparation, and revision. All authors read and approved the submitted version.
